# Intracavernosal Botulinum Toxin Injection for Erectile Dysfunction: A Comprehensive Systematic Review

**DOI:** 10.3390/life15121826

**Published:** 2025-11-28

**Authors:** Vanessa Talavera Cobo, Carlos Andres Yanez Ruiz, Mario Daniel Tapia Tapia, Andres Calva Lopez, Carmina Alejandra Muñoz Bastidas, Francisco Guillen-Grima, Francisco Javier Ancizu Marckert, Luis Labairu Huerta, Marcos Torres Roca, Fernando Jose Diez-Caballero Alonso, Daniel Sanchez Zalabardo, Bernardino Miñana Lopez, Jose Enrique Robles Garcia

**Affiliations:** 1 Department of Urology, Clinica Universidad de Navarra, Av. Pío XII 36, 31008 Pamplona, Spain; vtalavera@unav.es (V.T.C.); cyanezruiz@unav.es (C.A.Y.R.); mdtapia@unav.es (M.D.T.T.); acalva@unav.es (A.C.L.); llabairu@unav.es (L.L.H.); jerobles@unav.es (J.E.R.G.); 2 Department of Urology, Clinica Universidad de Navarra, Marquesado de Santa Marta 2, 28027 Madrid, Spain; cmunozbasti@unav.es (C.A.M.B.); bminana@unav.es (B.M.L.); 3 Department of Preventive Medicine, Clinica Universidad de Navarra, Av. Pío XII 36, 31008 Pamplona, Spain; 4 Biomedical Research Center in Epidemiology and Public Health Network (CIBERESP), Carlos III Health Institute, 28029 Madrid, Spain; 5 Department of Health Sciences, Public University of Navarra, Av. Barañain, s/n, 31008 Pamplona, Spain; 6 Group of Clinical Epidemiology, Area of Epidemiology and Public Health, Healthcare Research Institute of Navarre (IdiSNA), 31008 Pamplona, Spain

**Keywords:** erectile dysfunction, botulinum toxin type A, intracavernosal injection, refractory, systematic review, PDE5 inhibitors

## Abstract

Background: Erectile dysfunction (ED) affects approximately 20% of men worldwide, significantly affecting their quality of life. While phosphodiesterase type 5 inhibitors (PDE5-Is) are the standard first-line treatment, a substantial number of patients are non-responders. Second-line treatments, such as intracavernosal alprostadil, are effective but often limited by their invasive nature and the need for frequent injections. Intracavernosal onabotulinumtoxinA (BoNT-A) offers a promising new option. By inhibiting acetylcholine release and norepinephrine, as well as other neurotransmitters involved in detumescence, it facilitates cavernosal smooth muscle relaxation and enhances penile blood flow. Its effects may persist for up to six months following a single injection, potentially reducing treatment burden and improving adherence among men with refractory ED. Methods: A systematic review was performed in accordance with the PRISMA guidelines. Literature searches were conducted in PubMed, Embase, Cochrane Library, Scopus, and Clinicaltrials.gov from inception until August 2025 using a combination of keywords and MeSH terms related to ‘erectile dysfunction’ and ‘botulinum toxin’. After screening, 51 studies met the inclusion criteria. Due to significant heterogeneity in interventions (e.g., BoNT-A dosage, co-therapies), patient populations, and reported outcomes, the data were not suitable for meta-analysis. Consequently, a narrative synthesis was performed to summarize the findings. Results: Among the included studies, intracavernosal BoNT-A was associated with improvements in validated erectile function scores. Reported response rates, variably defined across studies, ranged from 40% to 77.5%. Several studies suggested that efficacy was higher in patients with mild-to-moderate ED and with repeated administration of 100 U doses. The treatment exhibited a favorable safety profile. The most common adverse event was mild, transient penile pain (reported incidence 1.5–6%). No studies reported serious systemic adverse events. The overall strength of the evidence was limited by significant heterogeneity among the included studies and their generally small sample sizes. Conclusions: Based on this systematic review, intracavernosal onabotulinumtoxinA (BoNT-A) may be a beneficial therapeutic option for patients with refractory ED, offering potential improvements in sexual function while reducing the need for invasive therapies. Future large-scale, placebo-controlled studies are essential to confirm these benefits and standardize their clinical application.

## 1. Introduction

Erectile dysfunction (ED) is a prevalent global health issue, affecting approximately 20% of men worldwide, with its incidence rising progressively with age. The condition markedly impairs quality of life, self-esteem, and intimate relationships. For over two decades, phosphodiesterase type 5 inhibitors (PDE5-Is) have served as the primary first-line therapy, offering a non-invasive and effective treatment for many patients [1,2]. Nevertheless, a significant clinical challenge persists, as a substantial subset of men, particularly those with severe vasculogenic, neurogenic, or diabetic ED, demonstrate inadequate responses to these agents [3,4].

This therapeutic gap necessitates consideration of second and third-line treatments [5]. Intracavernosal injections of vasoactive agents, such as alprostadil, are an effective second-line alternative [6]. However, their adoption is often limited by patient apprehension regarding needle use, the technical challenges of self-administration, risks including priapism, and the requirement for frequent, on-demand dosing, which may reduce spontaneity [7,8]. Consequently, many patients with refractory erectile dysfunction face a difficult choice between foregoing treatment or proceeding directly to penile prosthesis implantation, a definitive but invasive and irreversible option associated with its own risks and costs [9].

This unmet clinical need has driven the exploration of novel, longer-lasting therapeutic modalities [10,11]. Among these, low-intensity shockwave therapy (Li-SWT) and platelet-rich plasma (PRP) injections are currently under active investigation [12,13,14,15]. Concurrently, intracavernosal administration of onabotulinumtoxinA (BoNT-A) has emerged as a particularly promising approach [16,17,18,19,20,21]. Three commercial formulations of BoNT-A are available: onabotulinumtoxinA, incobotulinumtoxinA, and abobotulinumtoxinA, and they are widely employed across various medical disciplines. BoNT-A, a potent neurotoxin, mediates its effect by inhibiting the presynaptic release of acetylcholine and other neurotransmitters at adrenergic nerve terminals within the corpus cavernosum. This results in prolonged chemical denervation, facilitating relaxation of cavernosal smooth muscle, reduction in sympathetic tone, and enhanced arterial inflow, thereby promoting erection [22,23]. A notable advantage of BoNT-A is its sustained duration of action, with clinical benefits reported to persist for several months following a single injection, potentially reducing treatment frequency and improving patient adherence [20].

Initial clinical studies, including several randomized controlled trials and meta-analyses, have reported encouraging improvements in erectile function scores (e.g., IIEF, SHIM, EHS) [2,3,4,5,7,9,13,22]. These studies consistently report a favorable safety profile, with adverse events predominantly limited to mild and transient local pain [7,24]. However, the current literature is marked by considerable heterogeneity in terms of study design, patient populations, BoNT-A formulations, dosing regimens, and outcome measures. This variability, compounded by the lack of large, definitive trials, leads to ongoing uncertainty regarding the precise efficacy, optimal clinical application, and positioning of BoNT-A within existing erectile dysfunction treatment algorithms [2,13,20].

Therefore, this systematic review aims to critically synthesize and evaluate the current evidence on the efficacy and safety of intracavernosal BoNT-A for the treatment of ED. By consolidating findings across studies, we seek to provide a clear summary of its therapeutic potential, identify factors predicting treatment success, and highlight key knowledge gaps to guide future clinical research [7,24].

## 2. Materials and Methods

### 2.1. Search Strategy and Study Registration

This systematic review was conducted in accordance with the Preferred Reporting Items for Systematic Reviews and Meta-Analyses (PRISMA) 2020 guidelines [25]. The review protocol was prospectively registered in the PROSPERO international prospective register of systematic reviews (Registration Number: CRD420251087894).

A comprehensive literature search was performed across multiple electronic databases, including PubMed, Embase, the Cochrane Library, Scopus, and ClinicalTrials.gov, from their inception until August 2025. To ensure search comprehensiveness, the AI-powered research platforms Consensus and Elicit were also utilized to screen for any additional relevant studies that may not have been captured by the database searches.

The search strategy was designed to incorporate a combination of relevant keywords and Medical Subject Headings (MeSH) terms. The core concepts included “Erectile Dysfunction,” “Botulinum Toxins,” and “Injections.” The following search string, adapted for each database, exemplifies the approach: (erectile dysfunction) AND (botulinum toxin OR Botox OR BoNT-A OR Xeomin OR Dysport) AND (intracavernosal injection OR penile injection).

### 2.2. Study Selection and Eligibility Criteria

Two independent reviewers screened the retrieved records by title and abstract, followed by a full-text assessment of potentially eligible studies. Any discrepancies between reviewers were resolved through discussion or by consultation with a third reviewer.

Studies were included if they investigated the use of intracavernosal botulinum toxin type A (BoNT-A) for the treatment of erectile dysfunction in human subjects. Randomized controlled trials, prospective and retrospective cohort studies, and case series with ≥10 patients were considered. Reviews, editorials, animal studies, and case reports with fewer than 10 patients were excluded.

### 2.3. Study Selection Process

**Figure 1:**
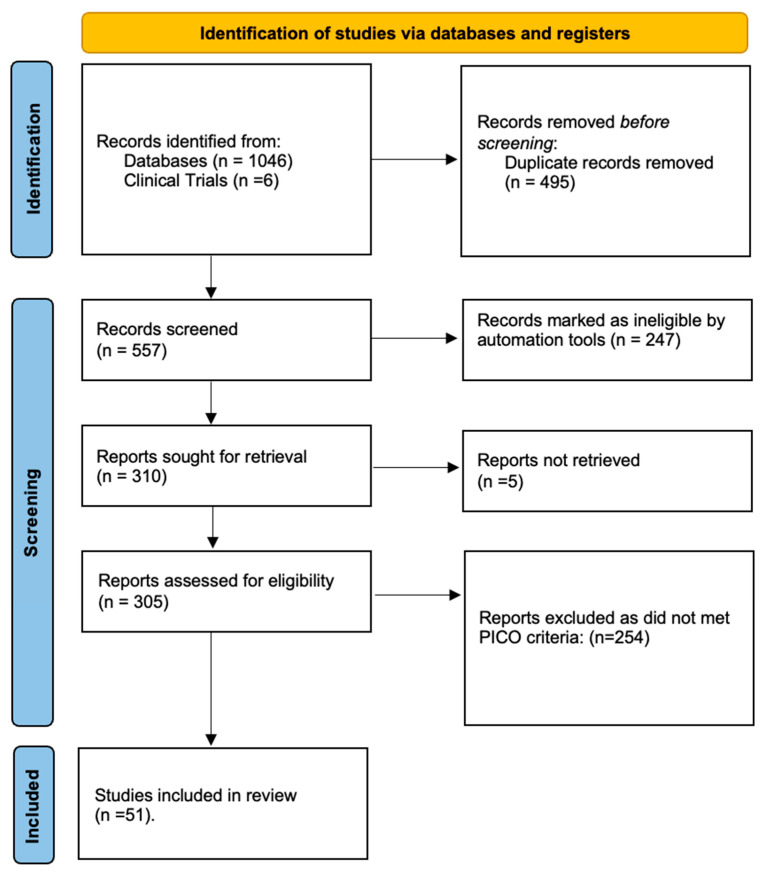
PRISMA 2020 flow diagram for new systematic reviews, which included searches of databases and registers.

The study selection process is detailed in the PRISMA flow diagram (Figure 1). A total of six relevant clinical trials were identified on ClinicalTrials.gov. Of these, results were publicly posted for three, and only one was associated with a peer-reviewed publication. Our initial database and register searches yielded 1052 records. After removing duplicates, 557 unique records were screened by title and abstract. Subsequently, 305 full-text articles were assessed for eligibility, resulting in 51 studies that met the inclusion criteria and were included in the final synthesis. To ensure a comprehensive capture of the literature, the reference lists of all eligible studies were manually screened.

### 2.4. Data Extraction

Data were systematically extracted from the included studies using a standardized data collection form. The extracted information included the following:

- Study characteristics: First author, publication year, country, and study design.
- Patient demographics: Sample size, mean age, and ED etiology and severity.
- Intervention details: BoNT-A formulation (e.g., onabotulinumtoxinA), total dose, number and frequency of injections, injection technique, and any concomitant ED therapies.
- Outcome data: As outlined below.

### 2.5. Outcomes

The primary outcome was the change in erectile function from baseline, measured by validated instruments, including the International Index of Erectile Function (IIEF), the Sexual Health Inventory for Men (SHIM), and the Erection Hardness Score (EHS). Where available, mean differences with standard deviations (SD) or 95% confidence intervals (CIs) were extracted.

Secondary outcomes included the following:

- The safety and adverse event profile, categorized by nature and frequency.
- The duration of the therapeutic effect.
- Changes in hemodynamic parameters as assessed by penile duplex ultrasonography.

### 2.6. Data Synthesis and Risk of Bias Assessment

Due to substantial clinical and methodological heterogeneity across the included studies in terms of design, patient populations, and outcome reporting, a quantitative meta-analysis was deemed inappropriate. Therefore, the findings were synthesized narratively.

The risk of bias for included randomized controlled trials was assessed using the Cochrane Risk of Bias tool (RoB 2, 2019 version), and the overall judgments were visualized graphically using the robvis (Risk-of-Bias Visualization, 2019 version) tool.

## 3. Results

### 3.1. Characteristics of Included Studies

The 51 studies included in this synthesis encompassed a range of designs, including randomized controlled trials (RCTs), systematic reviews, meta-analyses, retrospective case series, and pilot studies. The sample sizes of the primary clinical studies varied substantially, from small pilot cohorts (*n* = 15–70) to larger multicenter trials (*n* = 176). The systematic reviews and meta-analyses aggregated data from multiple primary studies [1,2,3,4,7,8], key papers are shown in Table 1. Most of the clinical studies focused on men with ED refractory to PDE5-Is, while a minority included broader ED populations. As pre-specified in the protocol, articles were excluded if they were not published in English, the full text was not accessible, or they did not involve human subjects.

### 3.2. Efficacy Outcomes

Intracavernosal BoNT-A injection was associated with significant improvements in erectile function across multiple studies. These improvements were consistently demonstrated using validated patient-reported outcome measures, including IIEF, SHIM, and EHS, as well as objective parameters from penile Doppler ultrasound [1,2,3,7,8,9]. The calculated response rates, defined as achieving a minimal clinically important difference in the IIEF-EF domain, ranged from 40% to 77.5%. Subgroup analyses indicated that higher efficacy was observed in patients with less severe ED and in those who received repeated injection cycles [1,2,3,12,22]. These findings are supported by meta-analyses, which confirm statistically significant improvements in erectile function scores compared to placebo, with a more pronounced effect noted particularly at the 100 U dose [1,7,8].

### 3.3. Safety and Adverse Events

The intracavernosal administration of BoNT-A was found to be generally well-tolerated. The most frequently reported adverse event was mild and transient penile pain or discomfort at the injection site, with an incidence ranging from 1.5% to 6% across studies [2,3,7,12,13]. Serious adverse events were rare, with only isolated case reports of priapism or localized tissue reactions [1,2,3,7,8,12,13]. Positively, no systemic side effects related to the toxin were reported in any of the included studies.

### 3.4. Predictors of Response and Durability

Analysis of the included studies identified several factors associated with improved and sustained treatment outcomes. The administered dose was a significant moderator of effect durability; higher doses of 100 U consistently demonstrated a longer duration of efficacy, maintaining therapeutic benefit for up to six months in certain cohorts, compared to lower 50 U regimens [7,23]. Furthermore, observational data indicate that repeated BoNT-A injection cycles may enhance and prolong the therapeutic response compared to a single administration [2,13]. Emerging evidence also suggests that objective biomarkers, such as penile shear wave elastography, may help identify patients with favorable tissue compliance who are most likely to benefit from the treatment, although this requires further validation [22].

### 3.5. Risk of Bias Assessment

**Figure 2:**
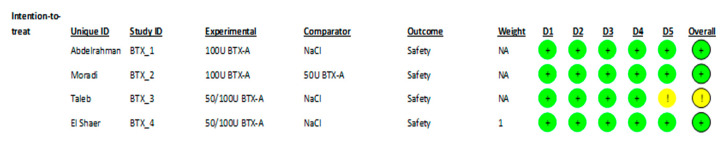
Risk of bias assessment for each parallel clinical trial included (RoB2). Green circles indicate “low risk of bias”; yellow circles indicate “some concerns” according to the RoB 2 assessment criteria. [4,7,23,24].

Methodological quality was appraised using the Cochrane Risk of Bias tool for randomized trials (RoB 2). A summary of the judgments for each domain across all studies is visualized in Figure 2. While most studies demonstrated low risk of bias in key areas such as random sequence generation and outcome measurement, a common area of concern was the lack of blinding of participants and personnel due to the interventional nature of the treatment.

## 4. Discussion

ED remains a highly prevalent condition, and the treatment of cases refractory to first-line therapies continues to pose a considerable clinical challenge. This persistent unmet need has driven the exploration of novel therapeutic strategies, among which intracavernosal injection of BoNT-A has emerged as a particularly promising intervention.

The basis for BoNT-A use lies in the underlying mechanisms of ED, which often include endothelial dysfunction, disrupted nitric oxide (NO) signaling, and excessive sympathetic activity, causing increased cavernosal smooth muscle contraction. BoNT-A counteracts this by inhibiting the presynaptic release of acetylcholine and, crucially, norepinephrine from adrenergic nerves within the corpus cavernosum [2,12,13]. This reduces sympathetic-driven vasoconstriction, promoting smooth muscle relaxation. The resulting improvement in penile blood flow and erection rigidity is especially relevant for patients with vasculogenic or neurogenic ED who have failed to respond to PDE5-Is or intracavernosal prostaglandins [2,12,13].

Moreover, the sustained efficacy observed with repeated injections suggests a prolonged neuromodulatory effect, reinforcing BoNT-A’s potential as a mechanism-based therapy that directly targets an underlying pathological component of refractory ED [13].

While botulinum toxin has a well-established role in urology for conditions such as overactive bladder, its application in ED represents a novel and promising frontier in sexual medicine [7]. This review synthesizes evidence from several key randomized controlled trials (RCTs), including four double-blind studies conducted by El-Shaer et al. [24], Moradi et al. [7], Taleb et al. [23], and Abdelrahman et al. [4], which form a critical part of the evidence base.

These four RCTs exclusively enrolled men with vasculogenic ED refractory to conventional medical therapy. The interventions varied in formulation and dose: three trials utilized onabotulinumtoxinA at doses of 100 U (Abdelrahman et al., *n* = 70) or a range of 50–100 U (Taleb et al., *n* = 45; El-Shaer et al., *n* = 176) [4,23,24]. In contrast, the study by Moradi et al. (*n* = 40) employed abobotulinumtoxinA [7]. A summary of these trials is provided in Table 2. Notably, none of these studies reported any systemic adverse effects, underscoring the localized action of the treatment [7,23,24,26].

Collectively, the current literature supports intracavernosal BoNT-A as a safe and moderately effective treatment for ED, particularly for patients who have not responded to standard pharmacological therapies (Table 3) [1,2,3,7,8,9,13,23,24,26]. The improvements in ED function scores are clinically meaningful for a substantial proportion of patients, and the safety profile is highly favorable, dominated by minor and transient local adverse events [1,2,4,7,12,13,23,24,26]. The evidence is particularly compelling for its use as an adjunct to PDE5-Is or prostaglandin E1 (PGE1) injections, potentially enhancing their efficacy [2,12,13].

Furthermore, real-world observational data from Giuliano et al. suggest that repeated BoNT-A injections may augment and prolong the therapeutic effect, indicating a potential benefit from multiple treatment sessions [2,13]. This is consistent with the drug’s proposed mechanism, whereby the induced relaxation of cavernosal smooth muscle directly improves penile hemodynamics and rigidity, an effect demonstrated across multiple studies in men with vasculogenic ED [1,2,3,7,8,9,13].

### 4.1. Limitations

Despite these encouraging findings (Table 3), this systematic review highlights several important limitations within the extant literature. The current evidence is constrained by the preponderance of studies with small sample sizes, significant heterogeneity in trial design and outcome measures, and short follow-up durations [1,8,20,26]. Furthermore, the predominance of positive outcomes raises concerns regarding potential publication bias, wherein negative or null results may be underrepresented.

These limitations necessitate a cautious interpretation of the results and underscore a clear need for larger, multicenter, randomized controlled trials employing standardized protocols. Such studies are essential to confirm efficacy, establish long-term safety, and define optimal dosing strategies [1,8,20,26]. Future research should also prioritize the identification of reliable predictors of treatment response, including the validation of imaging biomarkers such as shear-wave elastography [22]. Additionally, critical gaps remain in understanding the synergistic potential of BoNT-A when combined with PDE5 inhibitors and its comparative effectiveness against other second-line therapies, such as low-intensity shockwave therapy or platelet-rich plasma injections. The absence of direct head-to-head comparisons prevents a definitive assessment of BoNT-A relative position within the ED treatment algorithm [2,3,4,7,8,9,13,22].

### 4.2. Unresolved Questions

While the current evidence positions intracavernosal BoNT-A as a promising therapy for refractory ED, its pathway to becoming a standardized treatment is contingent upon resolving three fundamental areas of uncertainty, as outlined in Table 4. First, the long-term therapeutic landscape remains largely uncharted. There is a critical need for prospective studies with extended follow-up periods to definitively establish the durability of effect and the cumulative safety profile of repeated BoNT-A injections. This data is indispensable for therapy risk-benefit ratio over time and for shaping sustainable clinical guidelines. Second, to move beyond a one-size-fits-all approach, a concerted effort is required to identify and validate predictive biomarkers—whether derived from clinical characteristics, imaging modalities like shear-wave elastography, or molecular profiles. The ability to pre-emptively identify optimal responders would revolutionize patient selection, maximizing therapeutic success rates and avoiding unnecessary procedures. Finally, the current heterogeneity in dosing and technique underscores the necessity for rigorous, controlled trials to establish an evidence-based optimal dosing regimen and injection protocol. Standardizing these parameters is a crucial prerequisite for ensuring consistent, reproducible clinical outcomes and facilitating the widespread, reliable adoption of this treatment across diverse healthcare settings and patient populations.

## 5. Conclusions

Intracavernosal botulinum toxin (BoNT-A) injection represents a promising and well-tolerated therapeutic strategy for men with ED refractory to standard pharmacological treatments. Current evidence indicates it is a moderately effective intervention capable of producing clinically meaningful improvements in erectile function. These benefits likely extend beyond questionnaire scores, potentially enhancing sexual satisfaction, confidence, and overall quality of life. By offering a durable effect from a minimally invasive procedure, BoNT-A may also reduce the need for more invasive options like penile prosthesis implantation in selected patients.

However, the translation of this promise into routine clinical practice is constrained by significant evidence gaps. The existing literature is largely composed of small-scale, short-term, and methodologically heterogeneous studies, limiting the strength of conclusions and generalizability of findings. To advance this treatment paradigm, a critical need exists for larger, long-term, multicenter randomized controlled trials. These studies must prioritize establishing robust patient selection criteria, defining the durability of effect, refining optimal dosing and injection protocols, and validating predictive biomarkers. Furthermore, investigating the synergistic potential of BoNT-A in combination with PDE5-Is could yield strategies for optimizing outcomes. Until such high-quality data is available, BoNT-A should be considered an investigational therapy within the broader management algorithm for difficult-to-treat ED.

## Figures and Tables

**Table 1:** Comparison of key studies on BoNT-A penile injection for ED.

Key Papers					
Title	Author and Date	Study Design	Population	Key Result	Adverse Events
1. The effectiveness and safety of intracavernosal botulinum toxin injections in the management of erectile dysfunction: a systematic review and meta-analysis of clinical studies	K.H. Pang (2025) [1]	Systematic review and meta-analysis	Men with ED, various severities	40–77.5% response rate, significant IIEF/SHIM improvement	Transient penile pain, rare priapism
2. Safety and Effectiveness of Repeated Botulinum Toxin A Intracavernosal Injections in Men with Erectile Dysfunction Unresponsive to Approved Pharmacological Treatments: Real-World Observational Data	F. Giuliano et al. (2023) [2]	Retrospective case series	ED refractory to PDE5-Is/PGE1	77.5% response, increased with repeated injections	Mild pain, rare burn
3. Safety and Efficacy of Botulinum Neurotoxin in the Treatment of Erectile Dysfunction Refractory to Phosphodiesterase Inhibitors: Results of a Randomized Controlled Trial	I.F.S. Abdelrahman et al. (2022) [4]	RCT	ED refractory to PDE5-Is	Significant improvement in EHS, SHIM, SEP-2/3	No severe adverse events
4. Long Term Effectiveness and Safety of Intracavernosal Botulinum Toxin A as an Add-on Therapy to Phosphodiesterase Type 5 Inhibitors or Prostaglandin E1 Injections for Erectile Dysfunction	F. Giuliano et al. (2021) [12]	Retrospective, uncontrolled	ED non-responders to PDE5-Is/PGE1	41–50% response at 6 months	Mild pain only
5. Efficacy of Intracavernosal Injections of 50-Unit versus 100-Unit Doses of AbobotulinumtoxinA (Masport^®^) in Vasculogenic Erectile Dysfunction with Phosphodiesterase Type 5 Inhibitors Resistant	S. Moradi et al. (2022) [7]	Double-blind RCT	Vasculogenic ED, PDE5i-resistant	100 U more effective and durable than 50 U	Brief penile pain only

**Table 2:** Main RCTs about intracavernosal injection of BoNT-A.

Author (Year)	Country	Toxin Type	Dose (U)	Sample Size (N)	Population	Study Design	Main Findings
Taleb et al. (2019) [23]	Egypt	Onabotulinum toxin A	50–100 U	45	Refractory vasculogenic ED	Double-blind RCT	Dose-dependent improvement in erectile function.
El-Shaer et al. (2021) [24]	Egypt	Onabotulinum toxin A	50–100 U	176	Refractory vasculogenic ED	Double-blind RCT	Improvement in IIEF and SHIM scores; effect maintained up to 6 months.
Moradi et al. (2022) [7]	Iran	Abobotulinum toxin A	100 U	40	Refractory vasculogenic ED	Double-blind RCT	Improved penile rigidity and patient satisfaction.
Abdelrahman et al. (2022) [4].	Egypt	Onabotulinum toxin A	100 U	70	Refractory vasculogenic ED	Double-blind RCT	Significant improvement in IIEF and EHS.

**Table 3:** Key claims and supporting evidence identified in this paper.

Claim	Evidence Strength	Reasoning	Papers
BoNT-A penile injection improves erectile function in men with ED refractory to standard therapies	Strong	Multiple RCTs and meta-analyses show significant improvements in IIEF/SHIM/EHS vs. placebo	[1,2,3,4,7,8]
BoNT-A injection is safe, with only mild, transient adverse events	Strong	Consistent safety profile across studies, no systemic side effects, rare serious events	[1,2,3,12]
Higher doses (100 U) of BoNT-A are more effective and durable than lower doses (50 U)	Moderate	Dose-comparison RCTs and case series show greater and longer-lasting effects with 100 U	[7,23]
Repeated BoNT-A injections may enhance and prolong the therapeutic response	Moderate	Observational data show increased response rates with multiple injections	[2,13]
Imaging biomarkers (e.g., shear wave elastography) may predict response to BoNT-A	Moderate	Pilot studies suggest a correlation between tissue stiffness and treatment outcome	[9]
Long-term efficacy and safety of BoNT-A penile injection remain uncertain	Weak	Lack of large, long-term RCTs and standardized protocols	[1,8,20,26]

**Table 4:** Key open research questions for future studies.

Question	Why
What is the long-term efficacy and safety of repeated BoNT-A penile injections for ED?	To determine durability and cumulative risk, informing clinical guidelines.
Which patient characteristics or biomarkers best predict response to BoNT-A injection?	To optimize patient selection and maximize treatment benefit.
What is the optimal dosing regimen and injection protocol for BoNT-A in ED?	To standardize practice and improve outcomes across diverse populations.

## Data Availability

The data presented in this study are available from the corresponding author.

## Abbreviations

The following abbreviations are used in this manuscript:

ED	Erectile Dysfunction
PDE5-Is	Phosphodiesterase Type 5 Inhibitors
BoNT-A	Botulinum Toxin Type A
FDA	U.S. Food and Drug Administration
IIEF	International Index of Erectile Function
SHIM	Sexual Health Inventory for Men
EHS	Erection Hardness Score
CIs	Confidence Intervals
RCT	Randomized Controlled Trial
NO	Nitric Oxide
